# Mimicking Antigen-Driven Asthma in Rodent Models—How Close Can We Get?

**DOI:** 10.3389/fimmu.2020.575936

**Published:** 2020-09-30

**Authors:** Francesca Alessandrini, Stephanie Musiol, Evelyn Schneider, Frank Blanco-Pérez, Melanie Albrecht

**Affiliations:** ^1^Center of Allergy & Environment (ZAUM), Technical University of Munich (TUM) and Helmholtz Zentrum München, German Research Center for Environmental Health, Neuherberg, Germany; ^2^Molecular Allergology/Vice President's Research Group, Paul-Ehrlich-Institut, Langen, Germany

**Keywords:** mouse model, asthma, T2 airway inflammation, non-T2 airway inflammation, endotypes

## Abstract

Asthma is a heterogeneous disease with increasing prevalence worldwide characterized by chronic airway inflammation, increased mucus secretion and bronchial hyperresponsiveness. The phenotypic heterogeneity among asthmatic patients is accompanied by different endotypes, mainly Type 2 or non-Type 2. To investigate the pathomechanism of this complex disease many animal models have been developed, each trying to mimic specific aspects of the human disease. Rodents have classically been employed in animal models of asthma. The present review provides an overview of currently used Type 2 vs. non-Type 2 rodent asthma models, both acute and chronic. It further assesses the methods used to simulate disease development and exacerbations as well as to quantify allergic airway inflammation, including lung physiologic, cellular and molecular immunologic responses. Furthermore, the employment of genetically modified animals, which provide an in-depth understanding of the role of a variety of molecules, signaling pathways and receptors implicated in the development of this disease as well as humanized models of allergic inflammation, which have been recently developed to overcome differences between the rodent and human immune systems, are discussed. Nevertheless, differences between mice and humans should be carefully considered and limits of extrapolation should be wisely taken into account when translating experimental results into clinical use.

## Introduction

Asthma is a heterogeneous disease which affects around 300 million individuals of all age groups and its prevalence is increasing worldwide. Its impact is considered similar to other major chronic diseases such as diabetes or Alzheimer disease (1, 2). Asthma is defined by a history of respiratory symptoms such a wheeze, shortness of breath, chest tightness, cough and variable expiratory airflow limitation (3). A chronic airway inflammation leads to airway remodeling with hyperplasia of goblet cells and mucus hypersecretion, hypertrophy and hyperplasia of smooth muscle cells and lung fibrosis. Different asthma phenotypes have been described, which drove the development of the concept of asthma endotypes, where each endotype is a subtype of a disease condition and defined by a distinct pathophysiological mechanism, in contrast to the disease phenotype, which comprises the observable characteristics of the disease (4, 5). Generally, asthma can be separated in two main endotypes, a so-called Type 2 endotype, characterized by T-helper Type 2-high inflammatory response and a non-Type 2 endotype, whereby also mixed endotypes are not rare (6, 7). While asthmatic reactions can also be induced without exogenous triggers (8, 9), only antigen-driven asthma models will be discussed here.

Airway Type 2 immune responses are mainly mediated by eosinophils, mast cells, basophils, T2 cells, group 2 innate lymphoid cells (ILC2s) and IgE-producing B cells (10, 11). The whole inflammatory process starts with the activation of epithelial cells and release of cytokines such as IL-25, IL-31, IL-33, and TSLP which contribute to downstream T cells- and innate lymphoid cells-mediated T2 immune responses. These are characterized by the release of cytokines such as IL-4, IL-5, IL-9, and IL-13, consequent production of allergen-specific IgE, recruitment of eosinophils and other inflammatory cells, production of mucus and smooth muscle hyperreactivity (12). Non-Type 2 asthma, instead, is characterized by airway inflammation in the absence of eosinophils and is often associated with environmental and/or host hazards, such as cigarette smoke, pollution, work-related agents, infections, and obesity. These risk factors, alone or in conjunction, can activate specific cellular and molecular pathways leading to non-type 2 pulmonary inflammation (13). Growing evidence supports two major characteristic features of non-Type 2 asthma, namely a neutrophilic-driven inflammation and an IL-6-driven activation of the IL-17-dependent pathway (14, 15). To allow for a detailed investigation of molecular pathways critical for this complex disease or for a specific endotype in a functioning immune and respiratory system, many animal models of asthma have been developed, each of them trying to reproduce specific aspects of the human disease. Because of their low cost, high breeding efficiency and the large availability of transgenic models, rodents, and especially mice have classically been employed in asthma research, although considerations have been made regarding their limitations in mimicking human asthma (16, 17). In this review we will focus on antigen-driven asthma models and methods used for the elicitation and quantification of allergic airway inflammation, including lung physiologic, cellular and molecular immunologic responses. Furthermore, approaches to study exacerbations, chronicity and non-allergic airway inflammation as well as the value of humanized models will be discussed. Nevertheless, differences between mice and humans should be carefully considered and limits of extrapolation should be wisely taken into account when translating experimental results into clinical use.

## Eliciting Allergic Airway Inflammation

Historically, experimental asthma research was performed sensitizing rodents intraperitoneally with chicken ovalbumin (OVA) in combination with the pro-T2 adjuvant aluminum hydroxide (alum), followed by repetitive OVA exposures via the airways in order to elicit a Th-2 skewed adaptive immune response leading to eosinophilia, goblet cell hyperplasia and airway hyperresposiveness (18–20). Alum plays an important role in boosting the adaptive immune system via the inflammasome (21). The benefits of OVA lie on the fact that this substance is efficient, inexpensive and has well-characterized MHCI and MHCII epitopes and moreover OT1 and OT2 T-cell receptor transgenic mice are available, which allow monitoring of OVA-specific immune responses in the airways (22, 23), making OVA a very good option for unraveling underlying mechanisms of the disease. However, OVA is not allergenic upon inhalation, therefore it has been more and more replaced by naturally occurring allergens which possess higher clinical relevance.

Allergens frequently used in sensitization protocols include the house dust mites (HDM) *Dermatopagoides pteronyssinus* and *farinae*, the fungus *Alternaria alternata*, cockroach and pollen extracts. The principle of sensitization and challenge remained the same as it was for OVA, but here the use of the adjuvant became dispensable. Adjuvant-free models have been established using several intranasal instillations of these allergens, mimicking the natural exposure to airborne allergens via the nasal mucosa and airway tract (24–27). Some of these allergen complexes like HDM are characterized by an intrinsic protease activity which favors the initiation of the allergic response, stimulating the production of interleukin-25 (IL-25), IL-33, and thymic stromal lymphopoietin (TSLP) from airway epithelial cells, which in turn activate both dendritic cells, promoting T2 responses (28), and local ILC2, leading to the increased release of IL-5, IL-9, IL-13, and amphiregulin (26). Some others, like birch, grass or ragweed pollen grains, do not only release allergens, but also proinflammatory and immunomodulatory lipids and adenosine, which act as critical co-factors in the development of lung allergic inflammation (24, 29).

Whereas models using allergen sensitization/provocation via the airways is reminiscent of the standard route of sensitization in asthma and hay fever, there is also compelling data on the relevance of cutaneous exposure in the development of pulmonary allergy along the lines of the so-called “atopic march” in which eczema precedes food allergies, asthma or hay fever (30). Mouse models have confirmed that repeated epicutaneous sensitization to protein (aero)-allergens leads to phenotypes of atopic dermatitis and to increased risk of allergic rhinitis, lung inflammation and airway hyperresponsiveness, where skin barrier dysfunction and TSLP expression from keratinocytes play essential roles (31–35).

Besides the pulmonary inflammation upon allergen exposure, exacerbations induced by other factors like viral and bacterial infections are a characteristic feature in the course of disease (36, 37). Here murine models of asthma have been especially useful to identify possible effects of infections with the development of the pathology. Particularly, influenza (38–40), rhinovirus (41, 42) and respiratory syncytial virus (38, 43) are important pathogens in the childhood that have been associated with exacerbations in asthma.

Haptens are also broadly used in rodent models to investigate exacerbation in airway inflammation. Studies with toluene diisocyanate (TDI), trimellitic anhydride (TMA), dinitrofluorobenzene (DNFB), and picryl chloride (PCL), allowed dissecting the hapten-induced allergy as well as the similarities and differences between the different compounds (44–47). Rodent models of DNFB, a powerful sensitizer of non-atopic asthma (47), have recently shown increased numbers of macrophages in bronchoalveolar lavage fluid (BALF), tracheal hyper-reactivity and a strong neutrophilic-based lung inflammation that could reflect characteristic features of non-atopic asthma in humans (46, 48).

## Quantifying Allergic Airway Inflammation

The physiological characteristics of asthma are mediated by a complex interaction between multiple effector cells and mediators.

The increased infiltration of inflammatory cells is determined by total and differential cell counts as well as measurement of inflammatory mediator content in the BALF or lung tissue (24, 49, 50). Upon allergen provocation, especially the role of eosinophils is shown to be indispensable for the development of allergic airway inflammation by mediating influx of T cell subsets [reviewed in (51)] into the lung (52, 53). For their release of pro-inflammatory mediators these cells are important contributors to pathophysiological changes, including airway epithelial cell damage, mucus hypersecretion and goblet cell hyperplasia which can be observed and quantified in histological staining of lung tissue (20, 54). In this context, eosinophils can be quantified by cell surface markers and by direct counting of stained cells in histological specimen (55).

Regarding the measurement of inflammatory mediators, tissue-based *ex vivo* cultures are another way to examine which cytokines are regulated in the development of airway inflammation and asthma and which cell type plays a decisive role in the concerned organs [reviewed in (56)]. As an alternative to the determination of cytokines in the supernatant of lung homogenates, stimulation of cells isolated from lung tissue or draining lymph nodes, by adding e.g., the allergen is used to evaluate the distinct cytokine patterns and to examine cell type specific responses more precisely (57, 58), allowing initial mechanistic conclusions about the observed phenotype.

As a hallmark of T2-driven allergic asthma, allergen-specific IgE responses are quantified in murine sera e.g., by means of ELISA (enzyme-linked immunosorbent assay) or functional cellular assays (59). Another factor to be taken into account in this context is IgG (and its subclasses), which are known to modulate inflammation via its receptors (FcγR) (60, 61). For example, antigen-specific IgG has been shown to improve allergic airway inflammation when signaling via FcγRIIB on DCs (62) and triggering different FcgR via certain IgG subclasses engage different pathways in murine IgE-independent anaphylaxis (63). Interestingly, similar mechanisms are discussed to take place in humans as well (64).

Airway hyperresponsiveness (AHR), defined as the predisposition of the airways to react excessively to bronchoconstrictor agents or to noxious stimuli, is an essential component of the asthma phenotype. The degree of AHR usually correlates with disease severity (65), and can be employed clinically for therapy management (66). AHR may not replace measurements of lung function such as FEV1, however it has been proposed to be included with other indices of lung function for asthma control (67). Similarly to spirometry in cooperative humans, lung function testing has been developed for rodents. Analysis of AHR in animal models is usually performed using one cholinergic agonist (methacholine, carbachol, histamine, serotonine), which act on the muscarinic receptor transduction pathway coupling to airway smooth muscle contraction (68). Measurement of AHR is usually performed shortly (24–48 h) after allergen challenge either in whole body chambers in conscious animals (body plethysmography) or in tracheostomized animals, using systems such as the Buxco® or the Flexivent®, with the agonist being either injected or aerosolized (24, 50, 69). Whilst the measurement of Penh (enhanced pause) using body plethysmography has lost acceptance in the scientific community (70), measurement of respiratory system resistance (RL) and dynamic compliance (DC) together with other physiologic parameters under mechanical ventilation in tracheostomized animals is often employed in asthma research (50, 71, 72). An increase in RL reflects both narrowing of the conducting airways and alterations in the lung periphery (distal airways and parenchyma). On the contrary, decreases in DC reflect only events occurring in the lung periphery. Therefore, if the response to an intervention is limited largely to RL, then a relatively proximal location is implicated for the effect, whereas a distinctive effect on DC is indicative of a more distal site of action. (73).

The limitation of this technique is based on the fact that it is only applicable in terminal experiments. This has been overcome by the use of oro-tracheal intubation technique, allowing for repetitive measurements in the same animals, which can be of advantage in longitudinal studies (74, 75).

## Non-Allergic Asthma Models

Since the non-allergic asthmatic phenotype occurs also in patients with severe, steroid resistant asthma and management of asthma evolves into precision medicine with therapies directed toward specific phenotypes/endotypes (76–78), proper models of these conditions are needed to facilitate research on adequate therapeutic options (79). In this regard, it was shown that a Th17-driven non-eosinophilic lung inflammation is insensitive to several treatment options including steroids, by using adoptive transfer of *in vitro* polarized antigen specific Th17 cells with subsequent pulmonary allergen application (80, 81). Manni et al. could create a mixed phenotype by adoptive transfer of T2 and Th17 cells enabling them to dissect contributions of the different cytokine pathways to distinct features of airway disease like mucus metaplasia or tissue inflammation (82). Microbial components like bacterial lipopolysaccharides (LPS) used as adjuvants in airway application of allergen have been proven to elicit a non-eosinophilic airway inflammation by triggering pathogen recognition receptors (PRR). Kim et al. could demonstrate that in such models the dose of LPS during sensitization plays a decisive role in shaping the resulting lung inflammation either toward eosinophilic (low dose LPS) or neutrophilic (high dose LPS) inflammation (83). Comparing this airway sensitization model to intraperitoneal allergen application (with alum) Wilson et al. could illustrate how different sensitization regimes lead to different molecular and phenotypical pattern in the resulting airway disease identifying a prominent role for Th17 in neutrophilic airway inflammation and AHR (84). Hadebe et al. demonstrated the importance of microbial triggers in airway immune responses via initiation of a non-allergic steroid-refractory airway inflammation by combining two different agents (LPS and beta-glucan) (85). A more sophisticated approach uses biolistic transfection of a plasmid containing the genetic information of the allergen via gene-gun, with targeted expression in dendritic cells ensured by a specific promotor, leading to a Th1/Tc1 driven inflammation depending on IFNγ that is sensitive to steroid treatment (86). Application of Poly I/C, a dsRNA analog mimicking a viral infection, in combination with an allergen results in a Th1-driven airway inflammation as well, offering the possibility to study the pathomechanism underlying virus-induced airway inflammation (87). Taking advantage of the possibility to shape the resulting airway inflammation by means of different sensitization regimes (using the same allergen: house dust mite), Tan et al. were able to directly compare transcriptomic lung profiles of eosinophilic, neutrophilic and mixed phenotypes enabling identification of molecular pattern that are linked to distinct inflammatory phenotypes (88).

Aspirin-exacerbated respiratory disease (AERD) is a common, severe variant of asthma, which affects 7–10% of all asthmatics and is associated with overproduction of cysteinyl leukotrienes (cysLTs) and respiratory reactions to drugs that block cyclooxygenase 1 (89). The pathophysiology has not been fully solved yet, but in order to model this disease deficiency or overexpression genetic animal models have been used presenting severe eosinophilic respiratory mucosal inflammation (90, 91).

## Chronicity and Remodeling

Most of the above-mentioned models focus on the development of symptoms after a short period of antigen exposure. While this has provided a broad range of information on causal and mechanistic effects on asthma, it usually cannot mimic characteristics like chronic inflammation of the airway wall, mucus production and remodeling (92–95).

To compensate that limitation, several methods applying allergen for a longer period of time have been established. This causes a protracted experimental window up to several months and in some cases, due to the continuous exposure to the allergen, leads to tolerance in the mice (96–101). The transgenic technology allowed the generation of mice with characteristics of chronic asthma and airway remodeling (102, 103). Furthermore, transgenic models allowed the identification of an important migration factor of DCs to the lung (104) and the role of IL-33 receptor suppressor of tumorigenicity 2 (ST2) in development of chronic asthma in mice by regulating ILC2s, mast cells, IL9 and IL-13 in the lungs (105). In addition, recent gene modification in mice allowed to identify for example the role of the potassium channels Kca3.1 in airway remodeling (106), and the regulatory role of semaphorin 3E (Sema3E) in inflammatory and remodeling responses in chronic asthma (107).

Recently, CRISPR/Cas 9, a gene disruption technology, allowed to knock-out/down several genes in associated with exacerbation, inflammation and remodeling in asthmatic diseases, identifying roles for these molecules in some pathophysiological features of asthma. For example, using the CRISPR/Cas 9 technology the transient receptor potential (TRP) 1, an ion channel was successfully knocked-out by Reese et al. They could demonstrate its role in the protection from airway inflammation in rats as well as in mice, suggesting TRP1A as a therapeutic target in asthma (108). In another study depletion of long non-coding RNAs (lncRNAs), particularly AK085865, led to reduction of the inflammatory response in a murine model of asthma, by modulating differentiation of innate lymphoid cells progenitor (ILCP) into ILC2s (109). CRISPR/Cas 9, because of its high target specificity, is a tool that could be of high importance in the understanding of the pathomechanisms of asthma and identification of novel therapeutic targets.

## Humanized Mouse Models

Despite the widespread use of mouse models for the evaluation of asthmatic diseases, there are restrictions when comparing components of the murine biology (e.g., the immune system) with those of the human biology (110). Humanized mouse models, that are immunodeficient mice engrafted with functional human (immune) cells, help to overcome some of these discrepancies. They have become an important pre-clinical tool for biomedical research, but to date only a small number of humanized mouse models are available in the research field of asthma.

Currently immunodeficient mouse strains for this purpose are often based on IL2rg^null^ mice, which lack a functional common gamma chain (γc) of the IL-2 receptor. This chain is not only part of the receptor complex for IL-2, but assembles with other chains to form receptors for IL-4, IL-7, IL-9, IL-15, and IL-21 as well, which are expressed on several cells of the immune system and signaling via these receptors is essential for homeostasis of these immune cells [reviewed in (111)]. Thus, the lack of γc results in absence of functional T, B, and NK cells.

The three most commonly used strains in humanized models are: the NSG mouse, the NOG mouse and the BRG or BALB/c-Rag2^null^ IL2rg^null^ mouse. BRG and NSG mice have no gamma chain while NOG mice have a truncated cytoplasmic domain of the gamma chain, preventing signal transmission (112, 113). All three models allow for efficient engraftment with human immune cells, due to a severe impairment in development of T and B as well as NK cells. These new models have enabled a multitude of new findings in the field of asthma research such as the interaction of allergen immunotherapy, clinical tolerance and cellular response, as well as new therapeutic options through the induction of peripheral tolerance by sGARP (glycoprotein A repetitions predominant) (114, 115). New mechanistic relationships were also clarified, such as the influence of the IL-33/IL-13 axis on the asthmatic airway inflammation or the anti-inflammatory effect of IL-35 in asthmatic diseases (116). Based on the immunodeficient IL2rg^null^ mouse, further mouse models emerged, including the Hu-SRC-SCID mouse and the BLT mouse as well as the Hu-PBL-SCID mouse providing further insight into our understanding of the development of AHR as a characteristic feature of allergic asthma (117) and discovery of new therapeutics, such as the use of TIM-1 antagonists as a possible treatment strategy for asthma (118).

## Limits of Extrapolation

Taken together, recent developments in asthma research led to a shift from solely applying allergic T2-driven eosinophilic airway inflammation models to a broader variety of airway inflammation models following the demand for precision medicine based on phenotype/endotypes in asthma management. However, it is important to be aware that, while the main hallmarks of asthmatic airway inflammation can be mimicked in such models, there are certain differences between mice and men which are reviewed in detail elsewhere (119, 120), that might limit translational impact of results obtained in mouse models. Some of these differences include immunological features (121, 122), which might be overcome by using humanized models, whereas others like anatomical discrepancies [e.g., lung anatomy, cell composition in the airways (123, 124)] will still differ in humanized mice. Moreover, the course of disease and treatment can often not be mimicked: asthma often begins in childhood when the lung is not fully developed yet, whereas experiments are mostly done in mice which do not spontaneously develop asthma, using adult animals with fully developed lung structure. Since the immunological response is shaped not only by the route, but also the amount and frequency of allergen exposure (23, 125, 126), a model that efficiently results in allergic airway inflammation might not necessarily mimic allergen exposure as it is experienced by the patients. Direct extrapolation of efficacy for therapeutic interventions obtained in mouse models is hampered by the fact that mouse models are conducted under highly controlled conditions (e.g., under specific pathogen-free conditions) which substantially affects the diversity of intrinsic and acquired immune responsiveness and may cause substantial immunological differences between these models and human (127, 128). Moreover, experiments are usually performed in genetically similar animals, which do not reflect the heterogeneity of asthmatic patients. To sum this up there is not the “one asthma model” mimicking human disease, but there is a huge variety of different approaches that allow to closely reproduce certain aspects of this complex syndrome with certain advantages and disadvantages (Table 1), enabling researchers to examine a scientific question from several different angels in order to contribute mosaic pieces for better understanding asthma.

**Table 1:** Advantages and disadvantages of T2-driven asthma mouse models.

**Mouse model**	**Advantages**	**Disadvantages**
OVA models	High efficiency, reproducibility, low cost Well-characterized MHCI and MHCII epitopes OT1 and OT2 T-cell receptor transgenic mice can be used to study OVA-specific immune responses in the airways	Adjuvants are needed for sensitization Provides good mechanistic insights, but no clinical relevance
Aeroallergen models	Do not need adjuvants Mimic natural exposure to airborne allergens via nasal mucosa and airway tract	Need several consecutive applications of allergens Amount of allergen exposure might not reflect natural exposure of patients
Epicutaneous sensitization models	Allow studies on atopic march Mimics physiologic condition of repeated skin exposures to allergens	Needs intradermal applications of allergen or damaged skin barrier
Chronic models of asthma	Allows the study of a chronic phenotype as frequently observed in asthma patients Allows to investigate lung tissue remodeling	Longer duration of experiments with frequent allergen applications Risk of tolerance induction
Transgenic models	Allows evaluating the role of particular cells, receptors or mediators in asthma pathophysiology Helps evaluating disease development/progression	The genetic modification can affect other phenotypes in the model Challenges in translating murine biology in human biology
Humanized models	Help to mitigate the inherent differences between mouse and humans that limit translation of the findings	Paucity of humanized mouse models for asthma research Anatomical discrepancies between mice and humans (e.g., lung anatomy, cell composition in the airways)

## Author Contributions

FA and MA conceived topic and structure of this mini review. FA, SM, ES, FB-P, and MA wrote the review. FA, FB-P, and MA reviewed the manuscript. All authors contributed to the article and approved the submitted version.

## Conflict of Interest

The authors declare that the research was conducted in the absence of any commercial or financial relationships that could be construed as a potential conflict of interest.
